# Editorial: SARS-COV-2 infections within inborn errors of immunity populations

**DOI:** 10.3389/fimmu.2023.1164045

**Published:** 2023-03-09

**Authors:** Leif Gunnar Hanitsch, Anete S. Grumach, Ankur Kumar Jindal

**Affiliations:** ^1^ Institute of Medical Immunology, Charité-Universitätsmedizin Berlin, Corporate Member of Freie Universität Berlin and Humboldt Universität zu Berlin and Berlin Institute of Health, Berlin Institute of Health (BIH) Center for Regenerative Therapies, Berlin, Germany; ^2^ Faculdade de Medicina, Centro Universitario Saude ABC, Santo Andre, Brazil; ^3^ Allergy Immunology Unit, Department of Pediatrics, Advanced Pediatrics Centre, Postgraduate Institute of Medical Education and Research, Chandigarh, India

**Keywords:** IEI, COVID-19, SARS-CoV-2, infection, T cellular immunity, antibody response, humoral immunity

Since the beginning of the COVID-19 pandemic, physicians and scientists working in the field of inborn errors of immunity (IEI) have been challenged with the management of this vulnerable group. The development of vaccines to fight against the pandemic was surprisingly fast, with an evident change in the prognosis of the disease. Nevertheless, the risk assessment for severe disease courses and management of SARS-CoV-2 infections within the IEI population was uncertain. Besides, how would the community evaluate the effect of vaccination in IEI patients? Newly discovered observations on humoral and T- cell immune response to COVID-19 vaccination kept being challenged with the emergence of new virus variants.

This special series on SARS-COV-2 infections in patients with inborn errors of immunity comprises six original studies. First, Nielsen et al. report on humoral immune responses in a cohort of CVID patients after receiving a third and fourth COVID-19 vaccination, showing an increase in specific antibody levels after the third vaccination. Specific antibodies tended to increase after the fourth vaccination, but booster vaccinations did not increase the frequency of seroconversion. No passive immunity was offered by Immunoglobulin therapy. A second study that focuses on the immunogenicity of the COVID-19 booster vaccination was conducted by Leung et al. This study provides important data on mRNA and inactivated COVID-19 vaccines in IEI patients after booster immunization and shows its effect on cross-neutralization against the SARS-COV-2 Omicron variant. However, a heterogeneous population was evaluated regarding the types of IEI and the number of vaccinations.

García-García et al. collected data on acute and long-term humoral immunity and T- cell immune responses to SARS-COV-2 infection in unvaccinated children and young adults with IEI. They report a higher incidence of COVID-19 pneumonia in IEI patients than in the age-matched general population but a weaker long- term immune response.

Shields et al. report on the impact of COVID-19 vaccination on hospitalization and mortality in patients with primary and secondary immunodeficiencies and observed that there were fewer infected IEI patients in comparison with the general population during the beginning of the pandemic, possibly related to the isolation and the fear of getting COVID-19 in this population. Also, high coverage of vaccination was reached in the IEI group.

The clinical relevance of vaccination was confirmed by Cousins et al. demonstrating the significantly increased risk of hospitalization in unvaccinated IEI patients in a large single- center study.

Last but not least, Milito et al. evaluated whether different national treatment approaches for acute COVID-19 affected clinical outcomes in a Dutch–Italian cohort of CVID patients. Data suggest that specific COVID-19 treatments, such as the use of antivirals and monoclonal antibodies, should be reserved for selected subgroups of CVID patients.

The reported studies contributed to several questions about the response of IEI patients to the disease and the vaccination, although their immunological condition would foresee a much worse prognosis for SARS-CoV-2 infection.

## Author contributions

All authors (AG, LH, and AJ) jointly contributed to the editorial and agree to be accountable for the content of the work. All authors contributed to the article and approved the submitted version.

